# Comparison of long non-coding RNAs, microRNAs and messenger RNAs involved in initiation and progression of esophageal squamous cell carcinoma

**DOI:** 10.3892/mmr.2014.2287

**Published:** 2014-05-30

**Authors:** SU-QING LI, FENG LI, YUN XIAO, CHUN-MEI WANG, LEI TUO, JING HU, XIAO-BIN YANG, JIN-SONG WANG, WEI-HONG SHI, XIA LI, XIU-FENG CAO

**Affiliations:** 1 Department of Surgical Oncology, Affiliated Nanjing Hospital and Oncology Center of Nanjing Medical University, Nanjing, Jiangsu 210006, P.R. China; 2 College of Bioinformatics Science and Technology, Harbin Medical University, Harbin, Heilongjiang 150081, P.R. China; 3 Department of Pathology, Affiliated Nanjing Hospital and Oncology Center of Nanjing Medical University, Nanjing, Jiangsu 210006, P.R. China

**Keywords:** long non-coding RNA, microRNA, messenger RNA, esophageal squamous cell carcinoma, canceration processes, bioinformatic analysis

## Abstract

Traditionally, cancer research has focused on protein-coding genes, which are considered the principal effectors and regulators of tumorigenesis. Non-coding RNAs, in particular microRNAs (miRNAs) and long non-coding RNAs (lncRNAs), have been widely reported to be important in the regulation of tumorigenesis and cancer development. However, to the best of our knowledge, investigation of the expression profiles of lncRNAs and a comparison of the involvement of lncRNAs, miRNAs and messenger RNAs (mRNAs) in esophageal tumorigenesis and development have not previously been performed. In the current study, intrinsic associations among the expression profiles of lncRNAs, miRNAs and mRNAs from normal esophageal tissues and those from cancer tissues were investigated. Oligonucleotide microarrays were used to detect the expression profiles of the three types of RNA in the canceration processes of human esophageal squamous cell carcinoma (ESCC) tissues. It was demonstrated that the different RNAs exhibit associated patterns of expression among normal esophageal epithelium, low-grade intraepithelial neoplasia (LGIN), high-grade intraepithelial neoplasia (HGIN), and carcinoma tissues, particularly in the critical period of canceration (HGIN to ESCC). Furthermore, the results indicated a high level of similarity in the potential function of lncRNAs, miRNAs and mRNAs in the processes of ESCC development. In the current study, a first generation atlas of lncRNA profiling and its association with miRNAs and mRNAs in the canceration processes of ESCC were presented.

## Introduction

**Figure 1:**
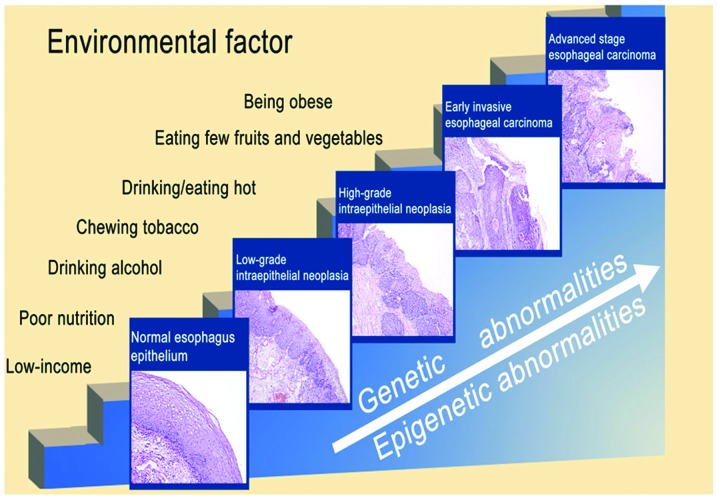
Initiation and progression of esophageal squamous cell carcinoma.

Esophageal carcinoma (EC) is one of the most lethal types of digestive tract malignancy according to 2011 cancer statistics, and there is clear geographic variation in its incidence throughout the world (1). Esophageal adenocarcinoma (EAC) and esophageal squamous cell carcinoma (ESCC) are the most common histopathological types of EC. EAC almost uniquely histopathologically features in western countries, but ESCC is the most common type in Asian countries such as China and Japan (1). Similar to the adenoma-carcinoma sequence of EAC (2), ESCC develops through progression from normal esophageal epithelium (NEE) to low-grade intraepithelial neoplasia (LGIN), high-grade intraepithelial neoplasia (HGIN), early stage esophageal carcinoma (EEC) and then advanced stage esophageal carcinoma (AEC) with an accumulation of genetic and epigenetic abnormalities (Fig. 1). Studies on esophageal precancerous lesions, including Barrett’s esophagus and EAC have produced valuable results (3) and a number of biomarkers have entered clinical trials in the USA (2). Other studies have also investigated precancerous lesions (3–5); however, further research is required. In China, a large-sample study demonstrated that the canceration rate in NEE was 1.4%, while the canceration rates of LGIN and HGIN were 5.8 and 38.9%, respectively (6). These results demonstrate that it is essential to identify novel molecular markers in precancerous patients that are at high risk of a malignant transformation in order to prevent the development of ESCC.

Historically, mild, moderate and severe dysplasia were the terms used to describe premalignant squamous epithelium cellular changes. Although it remains in use, this nomenclature has generally been replaced by the term of esophageal intraepithelial neoplasia (EIN), which is used to describe histological changes that are detected with biopsy. EIN, also known as esophageal precancerous conditions (EPC), is the potentially premalignant transformation and abnormal growth of squamous cells on the surface of the esophagus. Previous studies have confirmed that EIN was a definite risk factor for ESCC (4,5,7). Several novel genes associated with EIN and their molecular mechanisms of suppression, or even activation, have been detected. Kamangar *et al* (8) demonstrated that serum pepsinogen I (PGI) had no statistically significant association with EIN, whether analyzed as a dichotomous, ordinal (quartiles), or continuous variable, but a lower serum PG I/II ratio was linearly associated with higher risk of EIN. Chen *et al* (9) also confirmed that serum matrix metalloprotease-9 had a statistically different distribution between no dysplasia (normal and esophagitis) and dysplasia/early cancer subjects, yet this biomarker exhibited poor performance in a subsequent screening test and displayed low sensitivity and specificity. In cancerous tissues and precancerous lesions, Kobayashi *et al* (5) showed that p53 point mutation was involved in esophageal carcinogenesis. Another study reported that cyclin D1 overexpression starts early in dysplasia and could be a useful marker for its malignant potentiality, while reduction of p16INK4 and p27KIP1 expression occurs during the transformation from dysplasia to cancer (10). These findings suggested that numerous genes are abnormally expressed in EIN, which should be treated as a precancerous lesion. A previous study demonstrated that Ki-67 and ProExC can be used as an adjunct tool for diagnosing difficult cases of EIN (11). Another previous study showed that reduction of NOTCH1 expression directs the basal cells to cease terminal differentiation and to form an immature epithelium, thereby exhibiting a major role in the histopathogenesis of squamous epithelium neoplasia (12), but its expression and function in esophageal epithelium has not been investigated to the best of our knowledge.

The majority of the transcriptional output of the mammalian genome has been confirmed to be non-protein-coding (13), and these abundant parts of the transcriptome, which were previously regarded as ‘transcriptional noise’, have been identified to have important regulatory potential in transcription and post-transcription (14,15). Non-coding RNA (ncRNA) is a type of RNA that does not code for protein but has enzymatic, structural or regulatory function (16). ncRNAs can be classed as either small or long ncRNA, based on their transcript length (17). The most studied class of short ncRNA is microRNA (miRNA), which is involved in the specific regulation of its target messenger RNAs (mRNAs) through the inhibition of post-transcriptional cleavage or translation (18). Studies have demonstrated the differential expression patterns of miRNAs in numerous types of cancer. MiR-92a (19), miR-103/107 (20), miR-21, miR143, miR145, miR-205 (21) and miR-296 (22), among others, have been confirmed to be involved in the development of ESCC. lncRNA is a novel class of ncRNAs that are >200 nucleotides in length. Despite no known protein-coding potential, these RNAs demonstrate a wide range of structural and functional roles in various processes, including imprinting control (23), cell differentiation (24,25), immune responses (26,27), tumorigenesis (28–31), memory (32) and determination of pluripotency of embryonic stem cells (33). Large-scale analyses of full-length cDNA sequences have detected numerous lncRNAs in humans (34,35) and mice (36), but only a small number of these nucleic acids have been well characterized functionally. A number of studies have produced data implying that the effects of lncRNAs and their mechanisms of gene expression and regulation may be much broader and more complex than those of miRNAs (37–39). The development of molecular-profiling techniques, such as cDNA microarrays and transcriptome sequencing, may facilitate the provision of gene panels that identify EIN- and ESCC-specific molecular patterns. In a previous study, the upregulation of the lncRNA, HOTAIR, which was originally discovered in breast cancer tissues, was demonstrated to promote cancer metastasis and predict poor prognosis in ESCC (40). However, there are few studies concerning the expression profiles and functions of lncRNAs in esophageal diseases (41,42).

In the present study, ncRNA and mRNA expression profiles in EIN and ESCC samples were compared with those in NEE tissues using microarrays. To the best of out knowledge, this is the first study to determine the expression patterns of genome-wide miRNAs, mRNAs and lncRNAs in canceration processes of ESCC by microarray.

## Materials and methods

### Sample collection

The present study was approved by the Institutional Review Boards of the Cancer Center, Affiliated Nanjing Hospital of Nanjing Medical University (Nanjing, China), and all subjects provided written informed consent. All samples were obtained from the Nanjing Hospital (Nanjing, China), between January and September 2011. Biopsy specimens (NEE, LGIN, and HGIN samples) were obtained via esophageal endoscopy. Two biopsy samples were collected at once; the first biopsy tissue sample was routinely sent for pathological diagnosis, and then the pathological results determined whether the second sample was suitable for study. Unstained or lightly stained areas following Lugol’s iodine staining were considered to be precancerous lesions. EEC and AEC tissues were procured following surgical resection. Biopsies and surgical specimens were then placed in RNase-free freezer tubes (1.2 ml; Corning, Inc., Corning, NY, USA) and snap-frozen in liquid nitrogen.

### Histopathological analysis

#### Terminology

NEE denotes normal esophageal squamous epithelium, with no esophagitis, basal cell hyperplasia, or other abnormal conditions. LGIN denotes a low- or moderate-grade lesion. It refers to mild/moderate atypical cellular changes which are confined to the lower third or the basal two-thirds of the epithelium. HGIN denotes a high-grade lesion. It refers to severe atypical cellular changes spanning more than two-thirds of the epithelial thickness, including full-thickness lesions. Carcinoma *in situ* is a pathological type of HGIN, but much more severe. EEC is the primary tumor, which is confined to the adventitia of the esophagus, with no metastasis to the lymph nodes or distant organs. AEC refers to a primary tumor that has spread beyond the adventitia of the esophagus, with metastasis present in the lymph nodes or distant organs.

#### Biopsy samples

Twenty-three patients underwent esophageal endoscopy, and their biopsy samples were diagnosed based on the World Health Organization International Classification of Diseases for Oncology (2010). Five cases of NEE, four cases of LGIN and two cases of HGIN were diagnosed. A number of the subjects were diagnosed with esophagitis (n=8), or basal cell hyperplasia (n=4) without any other diagnoses of greater severity, so these subjects were excluded from further analysis.

#### Surgical samples

Among the twenty patients from whom samples were collected, three patients were discovered to have adenocarcinoma or were undergoing preoperative treatment, and eight samples were classed as well and poorly differentiated, so were excluded from further analysis. After exclusions, nine patients who had undergone no preoperative treatment remained, including seven cases of EEC (moderate differentiation), and two cases of AEC (with moderate differentiation).

All samples were selected based on their pathological diagnosis and then reviewed by another pathologist to ensure correct diagnoses. The clinicopathological features of the 20 patients included in the study were reported according to the pathological tumor-node-metastasis classification of the International Union Against Cancer (Seventh edition). The 5 patients who displayed no evidence of disease were selected as controls and were matched to the patients by age, gender and ethnicity (Table I).

### RNA extraction and quality monitoring

Biopsy samples were subjected to RNA extraction using the RNeasy Micro kit (Qiagen, Valencia, CA, USA) according to the manufacturer’s instructions. TRIzol reagent (Invitrogen, Carlsbad, CA, USA) was used according to the manufacturer’s instructions with minor modification in order to extract total RNA from the surgical samples. The aqueous phase was subjected to 3 steps of acid phenol/chloroform purification to eliminate protein residues prior to isopropyl alcohol precipitation. The resulting RNA pellet was then dissolved in 5 or 40 μl diethylpyrocarbonate-treated water. The RNA integrity was evaluated with a NanoDrop ND-2000 spectrophotometer (Thermo Fisher Scientific, Wilmington, DE, USA) and standard denaturing agarose gel electrophoresis.

For the microarray, equal volumes of total RNA from each of the patients were pooled separately in accordance with the RNA concentration of each sample to form disease and control sample pools.

### miRNA and lncRNA-mRNA microarrays

#### miRNAs

The 5 groups of samples were separately labeled using the miRCURY LNA miRNA Hy3/Hy5 Power Labeling kit (208030-A; Exiqon, Woburn, MA, USA) and hybridized on the miRCURY LNA miRNA array (version 14.0; 5th Gen Human; Exiqon). After the washing steps, the slides were scanned using the Axon GenePix 4000B Microarray scanner (Molecular Devices, Sunnyvale, CA, USA). Scanned images were then imported into GenePix Pro 6.0 software (Axon; Molecular Devices) for grid alignment and data extraction. Replicated miRNAs were averaged and miRNAs with intensities >50 in all samples were selected for calculating the normalization factor. Expressed data were normalized using the median. Following normalization, differentially expressed miRNAs were identified through fold change filtering.

#### lncRNAs and mRNAs

A Human lncRNA array version 1.0 (12×135k; Arraystar, Shanghai, China), containing probes for 18,534 lncRNAs and 18,874 coding transcripts (collected from databases such as NCBI RefSeq, UCSC Known Genes, NRED, RNAdb and Ensembl), was used for detection. Total RNA (~5 μg) from each sample was used for labeling and array hybridization with the following steps: i) Reverse transcription with a SuperScript Double-Stranded cDNA Synthesis kit (Invitrogen); ii) double-stranded cDNA labeling with a NimbleGen One-Color DNA Labeling kit (Roche, Mannheim, Germany); iii) array hybridization using the NimbleGen Hybridization System (Roche), followed by washing with the Nimblegen Wash Buffer kit (cat. no. 05584507001; Roche); and iv) array scanning using the Axon GenePix 4000B Microarray scanner (Molecular Devices). Scanned images (TIFF format) were then imported into NimbleScan software (version 2.5; Roche) for grid alignment and expression data analysis. Additionally, hierarchical clustering was performed to present distinguishable mRNA expression profiling among samples.

### Bioinformatic analysis

All data were divided into three groups (lncRNA, miRNA and mRNA), and each group contained five stages. Stage 1 (NEE) is the normal control condition, while stages 2 (LGIN), 3 (HGIN), 4 (EEC), and 5 (AEC) were the disease stages. The period between stages 3 and 4 is the most important transition period, so the dysregulation and potential functions of miRNAs, mRNAs and lncRNAs in these two stages were carefully analyzed.

#### Data preprocessing

Expression values of miRNAs, mRNAs and lncRNAs that were expressed in the five stages were selected (Table 1, Supplementary Material, http://210.46.85.200/Supplemental-Material/download.jsp), and differential expression values of the three categories of RNA were identified by screening for genes that were differentially expressed in the different disease stages compared with the normal group (Table 2, Supplementary Material). Two-fold changes was selected as the minimum difference threshold. Two collections of differentially expressed genes were extracted: One consisted of those that were differentially expressed between the normal group and all of the four disease phases (intersection), and the other included all of the genes that were differentially expressed between the normal group and any of the disease stages (union) (Table 1–3, Supplementary Material).

#### Data analysis

The following aspects were analyzed: (i) The extent of overlapping expression between the two collections and the clustering of the union and intersection differentially expressed RNAs were evaluated based on their fold change. (ii) The differential expression patterns of lncRNA, miRNA and mRNA at stages 3 and 4 vs. the normal group. The following sets of data were used for this analysis: The lncRNAs which were downregulated in stage 3 and upregulated in stage 4 vs. the normal group; the miRNAs which were significantly upregulated in stages 3 and 4 vs. the normal group; and the mRNAs which were downregulated in stages 3 and 4 vs. the normal group. The latter two sets of data were used to analyze the association between these reduced mRNAs and upregulated miRNAs. (iii) The similarities of the potential functions of miRNAs, mRNAs and lncRNAs in the initiation and progression of ESCC. a) Similarities between the miRNAs and mRNAs. The clustering analysis of the union and intersection sets of differentially expressed miRNAs, and the target mRNAs of these miRNAs were acquired. mRNAs that had significantly downregulated expression levels in stages 3 and 4 vs. the normal group were selected for further analysis. Then, the mRNAs that were both targets of the miRNAs and downregulated in the microarray during this period were selected. All of these selected mRNAs were subjected to GO analysis, and then all related miRNAs underwent functional enrichment by DAVID (http://david.abcc.ncifcrf.gov). b) Similarities between the mRNAs and lncRNAs. The lncRNAs with significantly upregulated expression levels in both stages 3 and 4 vs. the normal group were selected for further analysis. The two neighboring mRNAs of a lncRNA were used to define the function of the lncRNA. All related mRNAs were subjected to GO analysis, and then all related lncRNAs underwent functional enrichment by DAVID. c) Similarities between the miRNAs, mRNAs and lncRNAs. Functional similarities between the miRNAs and mRNAs, and the lncRNAs and mRNAs were compared in order to determine common features of the three types of RNA in the canceration processes of ESCC.

## Results

### Identification of lncRNAs, miRNAs and mRNAs dysregulated during the progression of esophageal carcinoma

**Figure 2:**
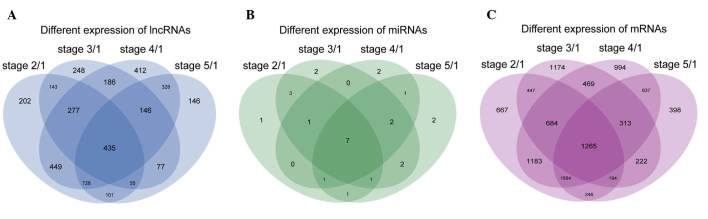
Venn diagrams presenting the differential expression of mRNAs, miRNAs and lncRNAs. Comparison of the numbers of (A) lncRNAs, (B) miRNAs and (C) mRNAs differentially expressed in each disease stage (stages 2–5) vs. the normal controls (stage 1). lncRNA, long non-coding RNA; miRNA, microRNA; mRNA, messenger RNA.

The detection rates of the expression of miRNAs, mRNAs and lncRNAs were 3.74 (59/1,700), 71.61 (13,517/18,874) and 77.14% (14,298/18,534), respectively. The results indicated that 4,404 lncRNAs, 36 miRNAs and 12,872 mRNAs were co-expressed in all five stages. Compared with the NEE (stage 1), the numbers of differentially expressed RNAs in the 4 disease stages (stages 2–5) were as follows: lncRNAs, 2,390, 1,567, 2,961 and 2,016; miRNAs, 15, 18, 14 and 17; and mRNAs, 6,370, 4,768, 7,229 and 4,959, respectively. The co-expressed and differentially expressed genes were compared, and 435 lncRNAs (Fig. 2A), 7 miRNAs (Fig. 2B), and 1,265 mRNAs (Fig. 2C) were expressed in all 5 stages and were differentially expressed in stages 2–5 vs. the normal group (Table 1–4, Supplementary Material).

### Similarities of lncRNAs, miRNAs and mRNAs in dysregulated processes of esophageal carcinoma

ESCC is characterized by its aggressiveness and poor prognosis, and frequently develops from varying degrees of IN (LGIN to HGIN), which is a premalignant pathological condition occurring in normal esophagi. A number of studies have confirmed that HGIN is the most common precancerous lesion and often advances to ESCC (3–12). Therefore, the present study focused on the HGIN to EEC period (stages 3–4), which is the most important transition period.

**Figure 3:**
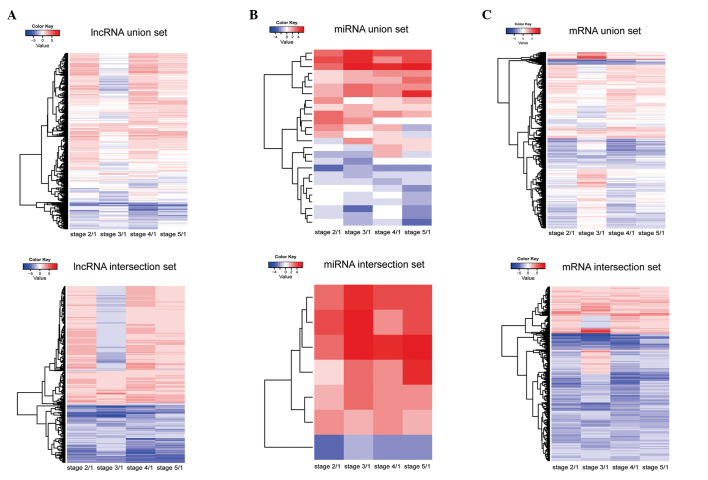
Microarray heatmaps presenting the differential expression of (A) lncRNAs, (B) miRNAs and (C) mRNAs in disease stages (stages 2–5) compared with normal controls (stage 1). Top diagrams represent the union set (all RNAs differentially expressed in at least one disease stage vs. the normal control), and bottom diagrams represent the intersection set (RNAs which were differentially expressed in every disease stage vs. the normal control). Blue represents downregulated genes, and red represents upregulated genes. lncRNA, long non-coding RNA; miRNA, microRNA; mRNA, messenger RNA.

Clustering analysis of the intersection (Fig. 3A, bottom) and union (Fig. 3A, top) sets of differentially expressed lncRNAs in the four stages of disease (compared with stage 1) were constructed based on the fold change. The results displayed that expression levels of the majority of lncRNAs were upregulated (Table 5, Supplementary Material).

Additionally, clustering analysis of the intersection (Fig. 3B, bottom) and union (Fig. 3B, top) sets of differentially expressed miRNAs in the 4 disease stages were constructed based on the fold change. In the clustering map of the union set of differentially expressed miRNAs, only 2 miRNAs (miR361-3p and miR1470) were significantly upregulated in stages 3 and 4.

### Similarities between miRNAs and mRNAs

miRNAs are endogenous small ncRNAs that are ~22 nt in length. They negatively regulate gene expression by binding to the 3′-untranslated regions (UTRs) of mRNA target transcripts, causing translational repression or mRNA degradation. As miR361-3p and miR1470 were indicated to be significantly upregulated in stages 3 and 4, the target mRNAs of these two miRNAs were analyzed, and the target data sets of the two miRNAs were obtained from target gene prediction databases, including TargetScan (www.targetscan.org), miRBase (www.mirbase.org) and miRanda (http://www.microrna.org). There were 5,247 target mRNAs of miR361-3p, and 526 target mRNAs of miR1470 (Table 6, Supplementary Material). Based on the fold change, clustering analysis of the intersection (Fig. 3C, bottom) and union (Fig. 3C, top) sets of differentially expressed mRNAs in the four stages of disease was performed. The expression analysis of mRNAs was somewhat indiscriminate, exhibiting patterns of upregulation and downregulation in stages 3 and 4. In order to study the association between miRNAs (increased expression in stages 3 and 4 vs. the normal group) and mRNAs, 2,943 mRNAs that were downregulated in stages 3 and 4 vs. the normal group were selected (Table 7, Supplementary Material). mRNAs that were both predicted targets of the miRNAs and downregulated in stages 3 and 4 were selected and identified as miRNA-associated mRNAs (intersection). A total of 642 target mRNAs of miR361-3p, and 71 target mRNAs of miR1470 were identified as miRNA-associated mRNAs (Table 8, Supplementary Material). All selected mRNAs in this intersection set underwent GO analysis, and 139 GO terms were acquired following functional enrichment analysis by DAVID (Table 9, Supplementary Material). According to the functional enrichment analysis of the mRNAs, 26 GO terms were involved in aspects of cancer, including the cell cycle, cell death, cell communication, signal transduction and apoptotic process. Results of the current study further supported the hypothesis that these dysregulated molecules may be involved in the complicated associations in ESCC development.

### Link between lncRNAs and mRNAs

The possible function of lncRNAs was probed according to neighboring (upstream and downstream) mRNAs in the current study. The neighboring mRNAs of lncRNA, which displayed significantly increased expression in stages 3 and 4 vs. the normal group were collected. In the present study, 1,887 lncRNAs with neighboring mRNAs were identified and all the related mRNAs underwent GO analysis and functional enrichment by DAVID (Table 10, Supplementary Material). The results indicated that these associated lncRNAs and mRNAs were involved in apoptosis, the cell cycle, proliferation, invasion and metastasis, which encompass the majority of the regulatory processes in the biological behavior of tumor cells.

### Functions shared by lncRNAs, miRNAs and mRNAs

**Figure 4:**
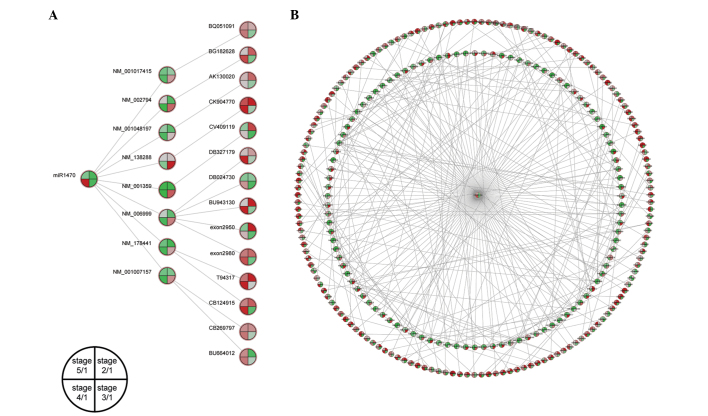
Cross-linked diagram of the similar potential functions among (A) miR1470 and (B) miR361-3p mRNAs and lncRNAs in the initiation and progression of ESCC. Green represents downregulated genes, red represents upregulated genes, and different quadrants represent different stages of the disease as indicated. mRNA, messenger RNA; lncRNA, long non-coding RNA; ESCC, esophageal squamous cell carcinoma.

**Figure 5:**
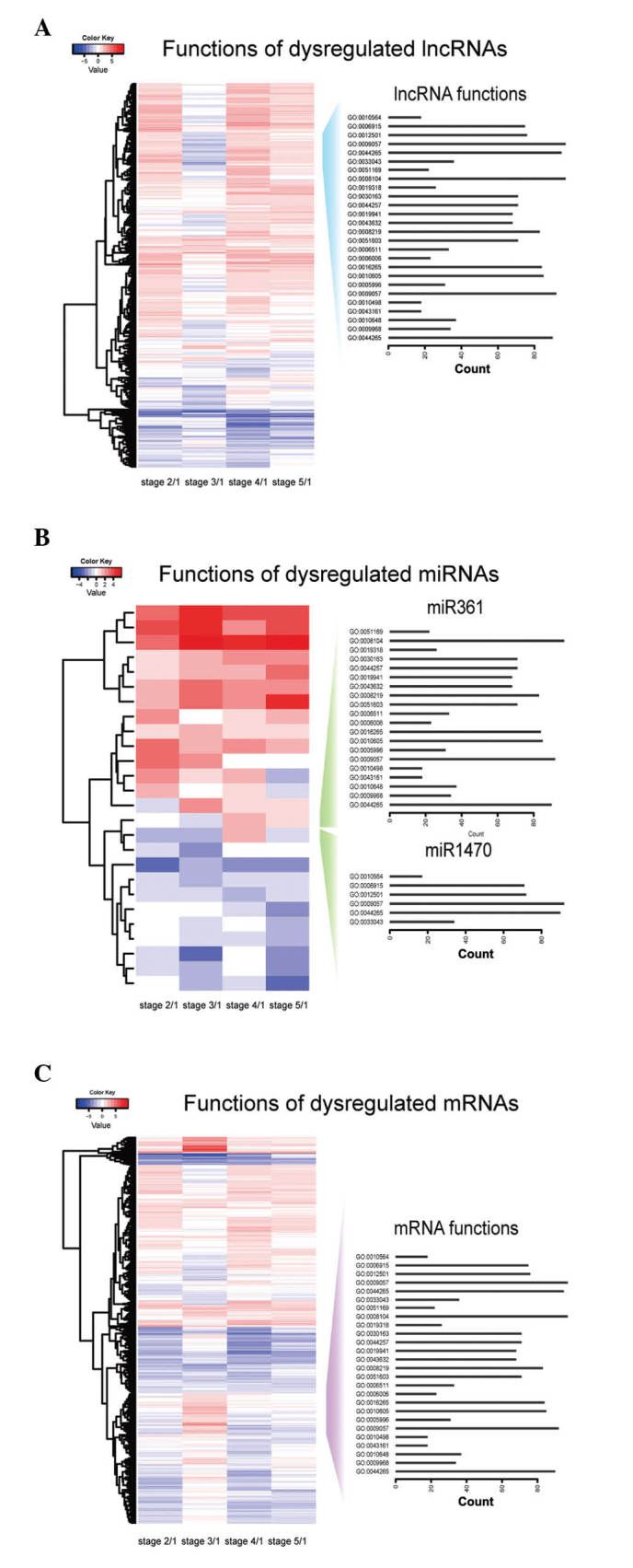
Functions of dysregulated (A) lncRNAs, (B) miRNAs (miR1470 and miR361-3p) and (C) mRNAs in the initiation and progression of ESCC. The dysregulated RNAs consist of those differentially expressed between the disease stages (stages 2–5) and the normal controls (stage 1). Blue represents downregulated genes, and red represents upregulated genes. lncRNA, long non-coding RNA; miRNA, microRNA; mRNA, messenger RNA; ESCC, esophageal squamous cell carcinoma.

The mRNAs that were both targets of miR361-3p or miR1470 and downregulated in stages 3 and 4 were obtained. Subsequently, the set of intersecting lncRNAs-mRNAs and miRNAs-mRNAs was selected and the similar potential functions among the miRNAs, mRNAs and lncRNAs in initiation and progression of ESCC were acquired (Table 11, Supplementary Material). Based on the analysis, the cross-linked diagrams of miR361-3p and miR1470 are depicted in Fig. 4; the three types of RNA shared similarities in the majority of the stages of disease progression. The function of these cross-linked lncRNAs, miRNAs and mRNAs in carcinogenesis and development of ESCC was then analyzed. The cluster analyses of the differentially expressed lncRNAs, miRNAs and mRNAs (disease stages vs. the normal group) are displayed in Fig. 5. The GO functional enrichment of the intersection set of mRNAs was then acquired. For example, the GO term 0008219 is correlated with cell death, and nine mRNAs (NM_171982, NM_022470, NM_145725, NM_000332, NM_001098517, NM_001004426, NM_152240, NM_002598 and NM_004394) that were downregulated in the disease and may be regulated by miR-361-3p were enriched in this GO term, while almost eight lncRNAs that neighbored the mRNAs were also enriched in the same GO term (Table 12, Supplementary Material). These results suggested that these lncRNAs, mRNAs and miRNAs may share similar potential functions in the regulation of cell death, possibly in apoptosis, cell cycle, invasion and metastasis, through which they promote the tumorigenesis and development of ESCC. Therefore, this study revealed the complicated interlinked functions of lncRNAs, miRNAs and mRNAs, and partially confirmed the regulation of the molecular network in ESCC (Table 12, Supplementary Material).

## Discussion

Malignant transformation from NEE to ESCC is a multistep process involving an accumulation of genetic and epigenetic changes. However, the mechanism of the development of ESSC remains unclear. Cell malignant transformation may be influenced by genetic background and environmental factors, including poor nutrition, unhealthy diet, smoking, drinking alcohol, and obesity. A previous study hinted that molecular changes may occur prior to histomorphological changes in the occurrence of cancer (43). The concept of the functional genome now includes a multitude of newly discovered classes of ncRNA transcript (16). Although the functional significance of ncRNAs has long been recognized, the abundance and scale of ncRNA (particularly lncRNA) expression changes in cancer is just beginning to become clearer. Thus far, few studies of the expression profiles and functions of lncRNA exist. It has been demonstrated that a novel lncRNA, HOTAIR was involved in various types of tumor. In a previous study, the lncRNA HOTAIR was upregulated and promoted cancer metastasis and predicted poor prognosis in ESCC (40). At present, the function of the majority of lncRNAs in ESCC is not clear. For this reason, charting the transcriptional landscape of coding and ncRNAs across normal and disease tissues is a key step in understanding the functional significance of transcriptome regulation in ESCC.

In the current study, to the best of our knowledge, an analysis of the stages of ESCC, human tissue-associated ncRNA and mRNA expression profiling was presented for the first time. Five lncRNA-mRNA microarrays and five miRNA microarrays were used to detect five NEE, four LGIN, two HGIN, seven EEC and two AEC tissues. However, the single size of each biopsy sample was too small to extract sufficient RNA to meet the requirements of the microarray, therefore, this study adopted a mixed-sample method, and the effective information of each individual could not be ascertained.

A first generation atlas of the expression profiles of coding and non-coding RNA has been produced in the present study, providing novel insight for this rapidly growing area of research into precancerous and cancerous diseases. Analysis in the present study indicated that 7 miRNAs, 1,265 mRNAs, and 435 lncRNAs exhibited differential expression between normal and disease tissues. The findings suggest that the majority of genes dysregulated during disease progression of ESCC influence the progression from the normal state to the IN and cancerous states. A high percentage of genes were dysregulated during the progression from HGIN to invasive cancer. The number of differentially expressed miRNAs was lower than that of mRNAs and lncRNAs in the stages 2–5, when compared with stage 1. It is also possible that the differences were due to the total number of miRNAs being less than those of mRNAs and lncRNAs. Although the total number of miRNAs in the intersection set was relatively small, it has been previously demonstrated that miRNAs are important in the regulation of the expression of their target mRNAs, thus confirming that miRNAs also have important roles in the tumorigenesis and development of esophageal diseases (44). In the present study, miRNAs, lncRNAs and mRNAs were demonstrated to be differentially expressed in the disease stages compared with the normal group, indicating that these types of molecules are involved in the tumorigenesis and development of cancer. It was also confirmed that a large number of the lncRNAs and mRNAs which were associated in the disease stages may be important in esophageal carcinogenesis. However, the distribution pattern of miRNAs did not correlate with that of protein-coding genes or lncRNAs.

Several studies have now indicated that lncRNAs regulate gene expression in cis or trans and may also function as transcriptional enhancers (45). Wamstad *et al* (46) hypothesized that if lncRNAs function in cis to regulate lineage commitment, then their neighboring genes should have functions associated with this process. To test this theory, they determined GO enrichment for the two nearest genes relative to the lncRNAs. Consistent with their hypothesis, they noted enrichment of genes involved in development, morphogenesis, and transcriptional processes. They found that lncRNAs identified in the data were significantly correlated in expression with their neighboring genes compared with randomly selected neighboring protein-coding genes. They tested the possibility that the observed correlations are attributable to coordinately regulated gene clusters; however, they observed that lncRNA expression is more highly correlated with the nearest adjacent gene (P=0.0275) relative to their background model.

Studies have demonstrated that lncRNAs perform their functions of gene regulation through interaction with their adjacent transcripts in cis. X-chromosome inactivation in mammals relies on XIST, a long non-coding transcript that coats and silences the X chromosome in cis. Vallot *et al* (47) reported the discovery of another lncRNA, XACT, which was expressed from and coated the active X chromosome specifically in human pluripotent cells, and in the absence of XIST, XACT was expressed from the two X chromosomes in humans but not in mice, suggesting a unique role for XACT in the control of human XCI initiation. Another paternally expressed lncRNA termed Kcnq1ot1 regulates epigenetic gene silencing in an imprinted gene cluster in cis over a distance of 400 kb in the mouse embryo, whereas the silenced region extends over 780 kb in the placenta (48). Furthermore, a large ncRNA called ANRIL (for antisense noncoding RNA in the INK4 locus) has been identified within the p15/CDKN2B-p16/CDKN2Ap14/ARF gene cluster. Genome-wide association studies also identified ANRIL as a risk locus for gliomas and basal cell carcinomas. Additionally, a mouse model has confirmed the pivotal role of ANRIL in regulation of CDKN2A/B expression through a cis-acting mechanism and its implication in proliferation and senescence (49). These results were consistent with the findings of Wamstad *et al* and further confirmed that it was sensible to probe the function of an unknown lncRNA based on the style of cis-regulation. Cis regulation is part of the mechanism of lncRNA gene regulation; however, thus far it has proven difficult to ascertain the cis or trans regulation manner of a specific lncRNA. In the current study, the cis analytical method was used, which partly revealed the function of lncRNAs in the initiation and progression of ESCC. According to the functional comparison of the association of lncRNA-mRNA and miRNA-mRNA, similarities in the potential functions of them in the carcinogenesis of esophageal squamous cells were identified. Consistent with this theory, methylation regulation (50) and ‘sponge adsorption’ theory (51), functional cross-linking among the three types of molecule has been demonstrated in the pathological process of cancer. Braconi *et al* (50) highlighted the inter-association between two classes of ncRNA, miRNA and lncRNA, and revealed miRNA-dependent regulation of lncRNA MEG3 expression by evaluating the involvement of miR-29, which can modulate DNMT 1 and 3. Their findings showed that overexpression of mir-29a increased the expression levels of MEG3. These data showed that methylation-dependent tissue-specific regulation of the lncRNA MEG3 by miR-29a may contribute to HCC growth. Wang *et al* (51) also demonstrated that the lncRNA HULC may act as an endogenous ‘sponge’, which downregulates miR-372 activity, and inhibition of miR-372 leads to reducing translational repression of its target gene, PRKACB, which in turn induces phosphorylation of CREB and HULC expression. Their data elucidated that fine tuning of the expression of the lncRNA HULC is part of an auto-regulatory loop, in which the inhibitory effect of HULC on the expression and activity of miR-372 allows upregulated expression of HULC in liver cancer. Collectively, these studies have identified a diverse repertoire of ncRNA functions but may have only scratched the surface of the functions of lncRNAs in cancer. Follow-up studies are required to reveal the related functions among lncRNAs, miRNAs and mRNAs in ESCC. Certainly, further regulatory mechanisms and related pathways remain undiscovered. Future studies should aim to pinpoint potential functions of lncRNAs and discern whether the non-coding genome contributes to ESCC, and the mechanisms by which it does this.

A total of twenty-six GO terms were observed in the present study, of which miR-361-3p and miR-1470 shared similarities in GO functional enrichment involved in cancer, including cell cycle, cell death, cell communication, signal transduction and apoptotic process. These miRNAs have been confirmed to have important roles in numerous types of tumor. miR-361-3p is a highly conserved X-linked miRNA that was demonstrated to be dysregulated in the serum of patients with lung cancer, and may be a blood-based marker for discriminating between malignant and benign lung tumors (52). Tanaka *et al* (53) reported that miR-361-3p was an oncogenic miRNA in human oral cancer cells. Hughes *et al* (54) confirmed that miR-361-3p expression was significantly increased in clear cell renal cell carcinoma compared with that in normal renal tissues. miR1470 has been demonstrated as another tumor-associated miRNA; Xiong *et al* (55) demonstrated that miR-1470 was one of known leukemia-associated miRNAs. A study by Sultan *et al* (56) also supported the theory by demonstrating miR-1470 overexpression in paclitaxel-resistant ovarian cancer cell lines compared with parental cell lines. The findings of this study provide novel insight into the carcinogenesis process of esophageal squamous cells, particularly regarding precancerous lesions. This study could also potentially be the basis of new predictive biomarkers in the future and aid improvement of the understanding of malignant transformation development.

Due to constraints of sample acquisition and research conditions, further experiments were not performed to validate the results of the present study. However, a number of previous studies have partially confirmed the results of this analysis. A number of the tumor-associated mRNAs that have been previously analyzed have been demonstrated to be involved in the initiation and progression of ESCC. For example, CDC25B was revealed to be an oncogene, influencing G2-M progression (57). Xue *et al* (58) analyzed the protein expression patterns of CDC25B and concluded that it would be valuable for the development of rational strategies for early detection of lesions that may lead to advanced ESCC. Furthermore, Li *et al* (59) demonstrated the expression levels of CDC25B in esophageal carcinoma to be significantly higher than those in dysplasia and normal tissues (48.1, 16 and 0%, respectively, P<0.01), and that CDC25B expression was correlated with the degree of differentiation and depth of invasion of tumor cells. Thus, CDC25B may be important in the early phase of ESCC. In addition, Jiang *et al* (60) demonstrated that the levels of ATM expression were increased in ESCC and premalignant lesions compared with those in normal tissues using *in situ* hybridization, and that increased ATM expression levels were associated with tumor de-differentiation. Their findings also suggested that the ATM gene should be further evaluated as a biomarker for the early detection of esophageal cancer in patients. DAPK1 is another associated gene that may participate in metastasis and apoptosis of ESCC cells, and its protein expression is closely correlated with the clinicopathological characteristics of ESCC (61). Currently, it is considered that EIN is a precancerous stage of esophageal cancer, and mounting evidence supports chronic inflammation as one of the promoters of EIN formation. BIRC2 was one of the inflammatory carcinogenesis-related genes in the present study. Daigeler *et al* (62) displayed that BIRC2 was upregulated in ESCC, while Fukuda *et al* (63) have reported that upregulated BIRC2 is associated with apoptosis and the inflammatory response. At present, EIN is considered to be a precancerous stage of esophageal cancer. Numerous studies have confirmed that chronic inflammation was one of the important factors promoting EIN formation. The aforementioned studies have shown that these dysregulated mRNAs may have important roles in the pathological process of ESCC. However, the regulatory mechanism of dysregulated mRNAs remains to be elucidated. Findings of the current study may provide novel insight into the processes of esophageal squamous cell carcinogenesis, particularly concerning precancerous lesions. Further studies are warranted to determine the functional role of these transcripts during the initiation and progression of esophageal tumors.

EIN is a potentially precancerous lesion, which is diagnosed histopathologically. Currently, only histological alterations are of practical value in routine clinical settings and histological diagnosis is the gold standard for surveillance of patients with IN. However, it is not presently possible to predict which lesions will progress. The combination of clinical parameters and genetic/epigenetic alterations increases the quality of the risk assessment of ESCC in IN patients. Identification of specific ncRNA patterns and their association with mRNAs in canceration processes of ESCC will help distinguish high-risk from low-risk patients. These potential biomarkers may be proteins or genes that can be differentially expressed in cancer, precancer and normal tissue.

The data of the present study demonstrate the similarities of lncRNAs, miRNAs and mRNAs in the initiation and progression of ESCC and provide novel insight for this rapidly growing area of research into precancer and cancer of the esophagus.

## Supplementary Materials

Supplementary Table 1.

Supplementary Table 2.

Supplementary Table 3.

Supplementary Table 4.

Supplementary Table 5.

Supplementary Table 6.

Supplementary Table 7.

Supplementary Table 8.

Supplementary Table 9.

Supplementary Table 10.

Supplementary Table 11.

Supplementary Table 12.

## Figures and Tables

**Table I:** Characteristics of patient tissues.

Sample number	Age	Gender	Pathological diagnosis
1	56	M	NEE
2	56	M	NEE
3	60	M	NEE
4	38	F	NEE
5	59	F	NEE
6	57	F	LGIN
7	69	M	LGIN
8	58	F	LGIN
9	59	F	LGIN
10	65	M	HGIN
11	52	M	carcinoma *in situ*
12	76	M	T3N0/20M0
13	62	M	T2N0/30M0
14	73	M	T3N0/11M0
15	69	M	T3N0/31M0
16	55	F	T3N0/12M0
17	59	F	T3N0/17M0
18	59	M	T3N0/18M0
19	60	M	T3N5/15M0
20	72	F	T3N1/33M0

NEE, normal esophageal epithelium; LGIN, low-grade intraepithelial neoplasia; HGIN, high-grade intraepithelial neoplasia; M, male; F, female.
